# The Molecular and Functional Characteristics of HLA-G and the Interaction with Its Receptors: Where to Intervene for Cancer Immunotherapy?

**DOI:** 10.3390/ijms21228678

**Published:** 2020-11-17

**Authors:** Jiji V. D. Attia, Charlotte E. Dessens, Ricky van de Water, Ruben D. Houvast, Peter J. K. Kuppen, Daniëlle Krijgsman

**Affiliations:** Department of Surgery, Leiden University Medical Center, P.O. Box 9600, 2300 RC Leiden, The Netherlands; J.J.V.D.Attia@lumc.nl (J.V.D.A.); C.E.Dessens@lumc.nl (C.E.D.); R.B.van_de_Water@lumc.nl (R.v.d.W.); R.D.Houvast@lumc.nl (R.D.H.); D.Krijgsman@lumc.nl (D.K.)

**Keywords:** HLA-G, immunotherapy, cancer, peptide presentation, HLA-G receptors

## Abstract

Human leukocyte antigen G (HLA-G) mediates maternal-fetal immune tolerance. It is also considered an immune checkpoint in cancer since it may mediate immune evasion and thus promote tumor growth. HLA-G is, therefore, a potential target for immunotherapy. However, existing monoclonal antibodies directed against HLA-G lack sufficient specificity and are not suitable for immune checkpoint inhibition in a clinical setting. For this reason, it is essential that alternative approaches are explored to block the interaction between HLA-G and its receptors. In this review, we discuss the structure and peptide presentation of HLA-G, and its interaction with the receptors Ig-like transcript (ILT) 2, ILT4, and Killer cell immunoglobulin-like receptor 2DL4 (KIR2DL4). Based on our findings, we propose three alternative strategies to block the interaction between HLA-G and its receptors in cancer immunotherapy: (1) prevention of HLA-G dimerization, (2) targeting the peptide-binding groove of HLA-G, and (3) targeting the HLA-G receptors. These strategies should be an important focus of future studies that aim to develop immune checkpoint inhibitors to block the interaction between HLA-G and its receptors for the treatment of cancer.

## 1. Introduction

Human leukocyte antigen G (HLA-G) is mainly expressed on the extravillous cytotrophoblasts in the placenta, where it mediates maternal-fetal immune tolerance during pregnancy [1]. While expression of HLA-G is restricted in healthy tissue, pathological conditions can induce HLA-G expression. In various cancers, de novo HLA-G expression has been observed, including in colorectal cancer (CRC), breast cancer, melanoma and ovarian cancer [2,3,4,5,6]. HLA-G expression is frequently associated with disease progression, tumor metastases, and poor clinical outcome [7,8]. Due to its immune-inhibiting functions, HLA-G is often claimed as an immune checkpoint in cancer [1]. Since the discovery of immune checkpoints, and their involvement in the pathogenesis of cancer, immunotherapies using monoclonal antibodies that block these checkpoints have been of great interest. The first immune checkpoint inhibitors that were approved by the US Food and Drug Administration (FDA) blocked the interaction between programmed cell death protein 1 (PD-1) and its ligand (PD-1L), and cytotoxic T-lymphocyte-associated antigen 4 (CTLA-4) and its ligands CD80 and CD86 [9,10,11]. This has led to remarkable therapeutic success in the treatment of multiple cancer types [9,10,11]. Various monoclonal antibodies against HLA-G have been developed, which are used for western blot, immunohistochemistry and flow cytometry [7]. The majority of the existing monoclonal antibodies directed against HLA-G do not recognize all HLA-G isoforms [12,13,14,15,16]. Additionally, cross-reaction has been reported of widely used HLA-G-recognizing antibodies with proteins other than HLA-G, such as classical HLA class I antigens [17,18]. Moreover, it is unsure if the existing antibodies can block the interaction between HLA-G and its receptors since the majority of the antibodies do not share the same binding site as HLA-G receptors [19]. Consequently, the existing monoclonal antibodies directed against HLA-G are not suitable for immune checkpoint inhibition in a clinical setting [17,18,20,21]. For this reason, it is essential that alternative approaches are explored to block the interaction between HLA-G and its receptors. This review discusses the molecular and functional characteristics of HLA-G and its receptors, and their interaction. Finally, research gaps and future directions for HLA-G-based cancer immunotherapy are discussed and proposed.

## 2. Structure of HLA-G

**Figure 1:**
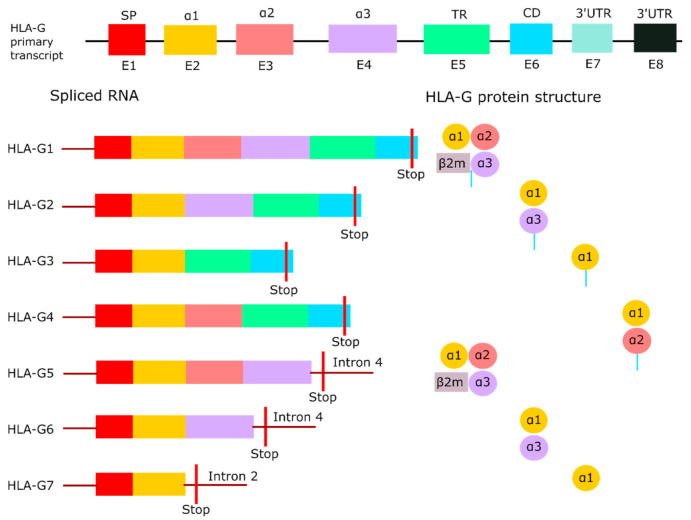
HLA-G isoforms. Overview of the full-length *HLA-G* primary transcript with its seven isoforms produced by alternative splicing. HLA-G1 to -G4 are membrane-bound due to the containment of the transmembrane region, while HLA-G5 to -G7 are soluble as a result of an early stop codon in intron 2 or intron 4. HLA-G1 and -G5 are the only isoforms that can bind the light chain β2M. Adapted from Krijgsman et al. [33]. Abbreviations: β2-microglobulin (β2M), cytoplasmic domain (CD), exon (E), human leukocyte antigen G (HLA-G), signal peptide (SP), three prime untranslated region (3′UTR), transmembrane region (TR).

Understanding the structure and assembly of HLA-G is crucial for the development of immune checkpoint inhibitors specifically binding to HLA-G. An important characteristic of HLA-G is the existence of different isoforms. The *HLA-G* primary transcript encodes seven different isoforms through alternative splicing [22], as illustrated in Figure 1. *HLA-G1, -G2, -G3* and -*G4* are membrane-bound, while *HLA-G5, -G6* and *-G7* are the soluble isoforms [10,23,24]. The full-length mRNA encodes *HLA-G1*. *HLA-G2* results from the splicing of exon 3, *HLA-G3* from the splicing of exon 3 and 4, and *HLA-G4* from the splicing of exon 4 [22]. All membrane-bound isoforms have a premature stop codon in exon 6. Exons 1 to 3 form the soluble *HLA-G5*, and exons 1, 2 and 4 form *HLA-G6* [22]. Both isoforms retained intron 4. *HLA-G7*, which is formed by exon 1 and 2, retained intron 2 as a result of incomplete splicing processes [25]. Intron 2 and 4 contain a forwarded stop codon, thereby preventing the transcription of the transmembrane domain [25].

Each exon of the *HLA-G* primary transcript encodes a part of the heavy chain of the protein. The heavy chain consists of a signal peptide (E1), three extracellular domains (E2 to E4), and a transmembrane domain with a cytoplasmic tail (E5 to E6) [24]. The extracellular domains are called α1, α2, and α3, with the latter noncovalently bound to the light chain β2-microglobulin (β2M) [23]. The peptide binding cleft is formed by the α1 and α2 domain, whereas the α3 domain serves as a binding site for co-receptors [26]. An unique characteristic of membrane-bound HLA-G is the truncated cytoplasmic tail due to the premature stop codon in exon 6. As a result, the protein lacks an endocytic motif, which prolongs the half-life of HLA-G, and reduces turnover compared to other HLA class I molecules [27]. HLA-G1 and -G5 are the only isoforms that can bind to β2M [28]. β2M functions as an additional binding site for receptors, but it has been demonstrated that HLA-G1 and -G5 do not necessarily need the association of β2M for binding to receptors [29], which will be discussed later. HLA-G isoforms are known to form homodimers via an intermolecular disulfide bond between the cysteine residues at position 42 in the α1 domain [30]. Only HLA-G3 cannot form homodimers, which indicates that the cysteine residue in HLA-G3 is not accessible for dimerization [31].

Most studies focused on HLA-G1 and HLA-G5, while studies investigating the other isoforms on protein level are lacking [32]. This is partly due to the absence of antibodies specifically targeting these other isoforms [20]. Only the antibodies 4H84, MEM-G1, and MEM-G2 are claimed to recognize all the HLA-G isoforms [16,17]. 4H84 and MEM-G1 have, however, been reported to cross-react with proteins other than HLA-G, including HLA-A2 [17,18]. Consequently, studying the presence of different isoforms in cancer using antibodies is not feasible at the moment. Combining multiple techniques can aid in the identification of the different isoforms by detecting HLA-G at various stages during protein synthesis, such as at RNA and protein level. Tronik-Le Roux et al. used immunohistochemistry and RNA sequencing to determine isoform expression in clear cell renal cell carcinoma [32]. This study discovered unannotated *HLA-G* transcripts with RNA sequencing, which indicates new isoforms. This suggests that the isoform repertoire of *HLA-G* is even more complex than previously thought. The question is whether these newly identified isoforms are also expressed on tumor cells at protein level. In order to investigate this, novel antibodies that detect these isoforms are required. Uncovering the HLA-G isoform repertoire expressed by tumors is essential to unravel their function and to develop immune checkpoint inhibitors specific for HLA-G-expressing tumors.

## 3. Peptide Presentation and Function of HLA-G

HLA class I molecules present endogenous peptide fragments on the cell surface to immune cells, thereby activating and regulating the immune response [34]. The classical HLA molecules HLA-A, -B and -C are highly polymorphic and present a wide range of peptides to T cells [35]. In contrast, nonclassical HLA molecules, which includes HLA-G, have limited polymorphism leading to a restricted peptide repertoire that can be presented [36]. The limited polymorphism can affect the capacity of HLA-G to bind receptors as the conformation and physical features of the presented peptide plays a crucial role in the receptor binding of HLA-G [37]. Therefore, studying the presentation of peptides by HLA-G can give more insights in the biological function of HLA-G and the interaction with its receptors, both in healthy and tumor tissue. This may also provide new approaches for immunotherapy to block the interaction between HLA-G and its receptors.

### 3.1. Peptide Loading Pathway

Elution of HLA-G peptides revealed a heterogeneous and complex mixture of peptides, which was less diverse compared to peptides derived from classical HLA class I molecules [38,39]. The majority of the peptides have a length of nine residues and are derived from intracellular proteins. Protein sources of the peptides include nuclear proteins, cytosolic proteins, ribosomal proteins, cytokine receptors, histones and, interestingly, tumor-associated antigens [38,39,40,41,42]. Peptides are loaded onto HLA-G in the endoplasmic reticulum (ER) by the peptide loading complex (PLC) formed by the transporter associated with antigen processing (TAP), HLA-G, the oxidoreductase ERp57, and the chaperones tapasin and calreticulin [43,44]. Both TAP and tapasin are important for HLA-G cell expression since inhibiting these proteins reduced cell surface expression [39,45]. While the larger part of the peptides is derived from TAP-dependent sources, HLA-G is able to bind peptides from TAP-independent sources as well [39,40]. These TAP-independent peptides have reduced affinity for HLA-G, suggesting that TAP is involved in the loading of high affinity peptides [46]. Tapasin seems to be involved in the same process, as HLA-G cannot bind high affinity peptides and is not transported to the cell surface when tapasin is inhibited [45]. HLA-G associated with low affinity peptides is not transported to the cell surface [45]. Instead, it is retrieved back to the ER and transported between the ER and the *cis*-Golgi until HLA-G is loaded with a high affinity peptide. In line with this observation, high affinity peptides prevent internalization of HLA-G1 and increase its cell surface expression [27]. Therefore, peptide loading is crucial for the regulation of HLA-G expression at the cell surface [23]. 

### 3.2. Primary Structure and Presentation of HLA-G1 Peptides

Over the years, efforts have been made to identify the peptides presented by HLA-G1. HLA-G1 peptides were first isolated from HLA-G-transfected lymphoblastoid cells (LCL) and analyzed by Lee et al. and Diehl et al. [38,39]. They determined the primary structure of the peptides through pool sequencing as summarized in Table 1. The majority of the peptides had a length of nine amino acids. The N- and C-terminus of the peptides consist of similar residues, whereas a variety of amino acids were observed in the central region of the peptides. Hydrophilic residues can be found in the central region, which is observed in many HLA class I ligand motifs [38]. While this was the first description of the primary structure of the peptides, later studies further analyzed HLA-G peptides with new techniques. Celik et al. described peptide position 1 as a tissue-specific anchor motif since two different HLA-G1 expressing cell lines showed a different amino acid preference at position 1 [47]. By defining the ligandome of the cell lines, it was revealed that the cell lines had similar protein source availability for peptide presentation. This indicates that the different amino acid preference at position 1 was the result of a difference in the structure of the binding pocket and not the available peptides in the cell. Thus, the structure of the binding pockets of HLA-G1 may differ per cell type, resulting in presentation of different peptides by HLA-G. Moreover, Di Marco et al. characterized peptide position 1 as an auxiliary anchor meaning that the residue contributes to a lesser extent to the binding to the peptide groove compared to primary anchor residues [41,48]. Lee et al. and Diehl et al. characterized peptide positions 2, 3 and 9 as primary anchor points; however, Di Marco et al. and Celik et al. did not regard peptide position 2 as a primary anchor point, implying that this position is less critical in binding of the peptide to HLA-G [38,39,41,47]. This discrepancy may be explained by the different cell lines and sequencing techniques used in these studies.

**Figure 2:**
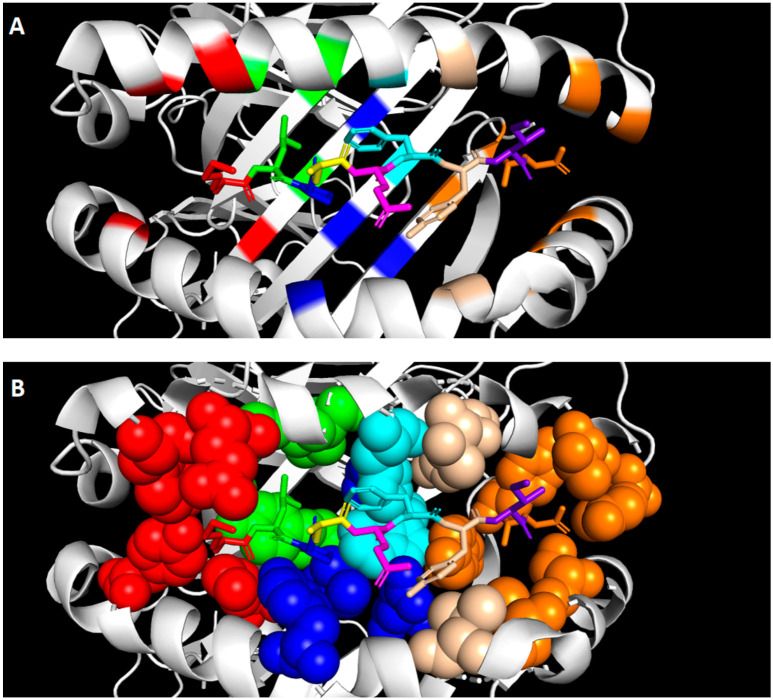
Primary structure and presentation of peptides presented by HLA-G1. The peptide is positioned in the peptide binding groove of HLA-G formed by two alpha helices and a beta sheet floor. Each position of the peptide with its corresponding binding pocket is visualized by different colors: position 1 and binding pocket A in red, position 2 and binding pocket B in green, position 3 and binding pocket D in blue, position 4 in yellow, position 5 in pink, position 6 and binding pocket C in cyan, position 7 and binding pocket E in beige, position 8 in purple, and position 9 and binding pocket F in orange. (**A**) The secondary structure of the binding groove is depicted with the peptide in the center. (**B**) The binding pockets are depicted as spheres to visualize the spacious structure of the pocket. The crystal structure was obtained from Walpole et al. [35] and modified with PyMOL (v. 2.4.0). Abbreviations: human leukocyte antigen G1 (HLA-G1).

Presented peptides by HLA-G1 lie in the peptide binding groove of HLA-G1, which is formed by two alpha helices and a beta sheet floor encoded by the α1 and α2 domain (Figure 2A). The peptide is positioned in an extended conformation in which half of the peptide is buried in binding pockets in the binding groove, which mediates the peptide binding. The other half is not anchored in a binding pocket (i.e., solvent exposed), and is involved in the binding of receptors [49]. Binding pockets anchor the peptide in HLA-G1 and each binding pocket occupies a peptide residue [50], as illustrated in Figure 2A,B. The structure of the binding pocket determines which amino acid it can occupy, thereby defining the primary structure of HLA-G peptides. Clements et al. analyzed the interaction between the presented peptides and the binding pockets of HLA-G [51], as summarized in Table 1. Position 1 of the peptide is partly anchored in the A pocket of HLA-G1 and is partly solvent exposed due to the hydrophobic residues found at this position. Thus, this peptide position mediates both peptide and receptor binding. It is suggested that this position is not involved in the direct binding of the peptide to the binding groove, but contributes to the stabilization of the peptide. This supports the description of position 1 as an auxiliary and tissue-specific anchor point [41,47]. The B pocket is a deep and hydrophobic pocket that holds the hydrophobic residues leucine, isoleucine or glycine at peptide position 2. Unique for HLA-G is the presence of the small serine in this area, which does not interact with the peptide in the B pocket but forms the bottom of the C pocket. As a result, the C pocket is deepened and can accommodate the larger aromatic residues at position 6 [38,51]. The D pocket is a shallow pocket harboring the small hydrophobic proline at peptide position 3. Another small hydrophobic residue is found at peptide position 7 in the narrow and deep E pocket. The C terminus of the peptide lies in the conserved F pocket. Positions 4, 5 and 8 do not lie in binding pockets, but are fully solvent exposed. These residues do not interact with the HLA-G binding groove.

The transport of HLA-G to the cell surface is only ensured when high affinity peptides are bound to the peptide binding groove of HLA-G. Tapasin ensures the loading of high affinity peptides onto HLA-G and the binding of the peptide to HLA-G is further monitored in the Golgi by quality control mechanisms [37]. The anchor points are crucial in this process as these are the main binding points of the peptide to the binding cleft [48]. The observation that cell lines can have different amino acid preferences suggests that the peptide repertoire that can be presented by HLA-G is depended on the tissue [47]. This is supported by the observation that the diversity of peptides derived from HLA-G expressed by placental tissue was different compared to the diversity of peptides from HLA-G-transfected cells [42]. Interestingly, 15% of the peptides from the placental tissue belonged to a cytokine-related protein, indicating specificity for a distinct peptide sequence. Furthermore, when HLA-G is transported to the cell surface after binding high affinity peptides, cell surface expression of HLA-G is prolonged due to the absence of an endocytic motif resulting in a decreased turnover rate of HLA-G. This can extend the availability of HLA-G at the cell surface and the time for immune cells expressing HLA-G receptors to bind to HLA-G. In summary, the peptides loaded onto HLA-G stabilize and prolong the expression of HLA-G, thereby enhancing its inhibitory capacities.

## 4. Binding Characteristics of HLA-G Receptors and Their Function

### 4.1. HLA-G Receptors: ILT2, ILT4 and KIR2DL4

**Figure 3:**
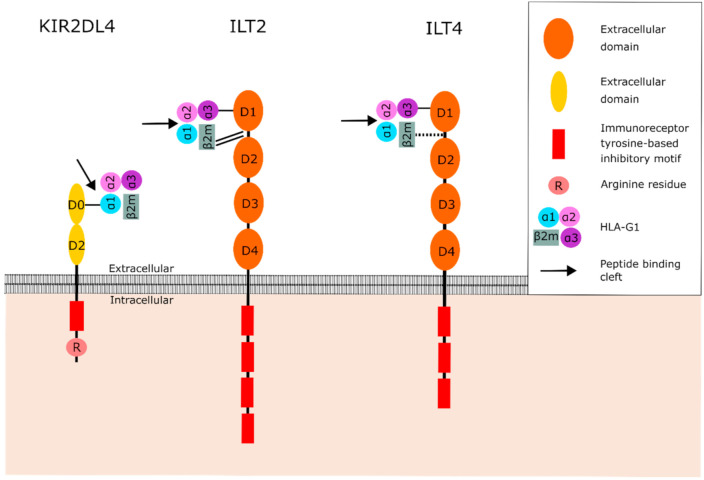
Structure of the HLA-G receptors KIR2DL4, ILT2 and ILT4, and their interaction with HLA-G1. The D0 domain of KIR2DL4 binds to the α1 domain of HLA-G1, which is part of the peptide binding cleft. However, the peptide is positioned deeper in the binding cleft than the position where KIR2DL4 binds to, suggesting that the peptide is not involved in the binding between KIR2DL4 and HLA-G1. ILT2 and ILT4 bind to the α3 domain and β2M, distal to the peptide binding cleft. The peptide is therefore not involved in binding between HLA-G and ILT2 or ILT4. The binding between the receptor and HLA-G is represented by black lines: single line (normal binding), double line (strong binding), dotted line (weak binding). Abbreviations: β2-microglobulin (β2M), human leukocyte antigen G (HLA-G), Ig-like transcript 2 (ILT2), Ig-like transcript 4 (ILT4), Killer cell immunoglobulin-like receptor 2DL4 (KIR2DL4).

HLA-G interacts with various receptors that originate from different receptor families. Understanding the structure and binding characteristics of these receptors is of great importance for the development of immune checkpoint inhibitors that hinder the function of HLA-G. Ig-like transcript 2 (ILT2) and 4 (ILT4) are members of the leukocyte immunoglobulin-like receptor (LILR) family. ILT2 is expressed on dendritic cells (DCs), B cells, NK cells and T cells, whereas ILT4 is restricted to cells of myeloid origin [52,53]. These ILT receptors have a broad specificity for HLA class I molecules; however, they have the highest affinity for HLA-G [54]. Both ILT2 and ILT4 contain four extracellular immunoglobulin domains (D1–D4), a transmembrane domain, and a cytoplasmic tail (Figure 3) [55]. The D1 and D2 domains are responsible for binding HLA-G [56,57,58]. The cytoplasmic tail consists of immunoreceptor tyrosine-based inhibitory motifs (ITIMs). Hence, ILT2 and ILT4 are known as inhibitors of the immune system [54]. ITIM signaling is mediated by Src homology-2-containing protein tyrosine phosphatase (SHP)-1 and -2 [59]. Liang et al. proposed a model in which ILT4 activation regulates DCs through recruitment of SHP-1 and -2 and activation of the NF-kB pathway, resulting in upregulation of IL-6 expression [60]. IL-6 can then activate the STAT3 pathway, resulting in impaired DC function and maturation. Signaling events downstream of ILT receptors in immune cells are not fully understood. Therefore, it is not known whether this proposed pathway can also impair the function of other immune cells.

Killer cell immunoglobulin-like receptor 2DL4 (KIR2DL4) belongs to the killer cell Ig-like receptors (KIR) and is expressed by NK cells [61]. KIRs recognize both classical and nonclassical HLA class I molecules. They are classified by the nature of their signaling domains where “L” (“Long” cytoplasmic tail) is inhibitory (e.g., KIR2DL) and “S” (“Short” cytoplasmic tail) is activating (e.g., KIR2DS). Moreover, they either contain two or three extracellular immunoglobulin domains (KIR2D or KIR3D) [62]. The inhibitory KIRs contain two ITIMs in their cytoplasmic domain, which recruit SHP-1 and -2 [59,62,63]. KIR2DL4 is different from the other KIR family members as it only contains one ITIM instead of two, and possesses an arginine in its transmembrane domain (Figure 3) [62,63,64]. Therefore, it is suggested that KIR2DL4 functions as both an activator and inhibitor of NK cells [62,63,64]. Indeed, both activating and inhibitory functions have been described for KIR2DL4 on NK cells [64]. KIR2DL4 signaling stimulates IFN-γ production and secretion by NK cells. In addition, to demonstrate that the single ITIM in KIR2DL4 is functional, a chimeric receptor of the 2DL4 cytoplasmic domain fused to the extracellular domains of KIR3DL1 was generated. Both activating and inhibiting domains were shown to be functional, suggesting that KIR2DL4 can either activate or inhibit NK cell function under different circumstances [63,65]. Although the single ITIM in KIR2DL4 is demonstrated to be functional, the downstream pathway of this single ITIM remains unknown. The structure of KIR2DL4 also differs from other KIR family members as the D1 domain is absent [62]. Instead, KIR2DL4 has a D0 and D2 domain as illustrated in Figure 3 [62]. KIR2DL4 is the only KIR that recognizes HLA-G, suggesting that the D0 or D2 domain of KIR2DL4 mediates the interaction between the receptor and HLA-G [62].

### 4.2. Binding between HLA-G and Its Receptors

The binding of HLA-G to its receptors is influenced by multiple mechanisms. It is shown that the binding affinity of HLA-G to ILT2 and ILT4 is heavily affected by its association with β2M, as illustrated in Figure 3 [56,57,66,67]. ILT2 interacts with HLA-G through the binding of the interdomain region (between D1 and D2) to β2M, and the D1 domain to the α3 domain [57,58]. In addition, ILT2 binds β2M with higher affinity than the α3 domain [57,58]. Therefore, ILT2 predominantly recognizes the β2M-associated HLA-G1 and -G5 isoforms [31]. In contrast to ILT2, ILT4 mostly relies on its binding with the α3 domain of HLA-G [58]. Consequently, ILT4 can bind β2M-associated and β2M-free HLA-G isoforms. KIR2DL4 binds to the α1-domain residues Met76 and Gln79, which are unique to HLA-G and present in all HLA-G isoforms [36,68,69].

In addition, dimerization affects the interaction between HLA-G and its receptors. As previously mentioned, all HLA-G isoforms with the exception of HLA-G3 can form homodimers. Dimerization of HLA-G improves the exposure to ILT binding sites by geometric configurational changes, resulting in increased HLA-G/ILT binding affinity [56,57,70,71]. Moreover, dimerization of HLA-G results in the availability of two accessible binding sites for its receptors, leading to binding of two receptors to one HLA-G dimer at the same time. This creates enhanced transmission of the inhibitory signal [70]. Furthermore, intracellular signaling is augmented by the HLA-G dimer due to the close position of the intracellular domains [58]. In contrast to the ILT receptors, KIR2DL4 is not able to bind to HLA-G homodimers due to the juxtaposition of two protomers, which are structural subunits of a protein complex, leading to steric clashes with KIR2DL4 [36]. Soluble HLA-G isoforms are predominantly monomeric, suggesting that KIR2DL4 might primarily bind to HLA-G5, -G6 and -G7 [36]. In addition, KIR2DL4 is undetectable on the cell surface of unstimulated NK cells, but only detectable intracellular. As a result of the small chance of interaction between KIR2DL4 and membrane-bound HLA-G in inactivated NK cells, KIR2DL4 might be recognized to a lesser extent by membrane-bound HLA-G isoforms, and to a higher extent by soluble HLA-G isoforms [71]. However, KIR2DL4 surface expression can be upregulated in activated NK cells, suggesting that KIR2DL4 can bind the membrane-bound HLA-G isoforms if present in a monomeric form [71].

Presentation of a diverse range of peptides is an essential hallmark of classical HLA class I molecules to induce a specific T cell response against various pathogens and diseases [34]. Due to its limited polymorphism resulting in limited protein variability and slow turnover, it is suggested that the primary function of HLA-G is not antigen presentation [26,72]. Since HLA-G only presents a restricted number of peptides, it is questioned how peptide presentation contributes to the biological function of HLA-G [36]. As illustrated in Figure 3, β2M and the α3 domain of HLA-G, which are involved in the binding to ILT receptors, are located distal to the peptide binding cleft. It is therefore unlikely that the peptide is directly involved in the binding of HLA-G to ILT2 or ILT4 [35,58]. KIR2DL4 does bind the peptide binding cleft; however, the peptide presented in the peptide binding cleft is expected to have no influence on the binding between HLA-G and the KIR2DL4 receptor since the peptide is positioned deeper in the peptide binding cleft than the position where KIR2DL4 binds to [36]. Thus, the interaction between HLA-G and its receptors is not influenced by the presented peptide. The presented peptide only seems to be important in the stabilization and prolongation of HLA-G on the cell surface.

Understanding the structure of HLA-G, ILT and KIR receptors is crucial when studying the interaction and function of HLA-G receptor signaling in cancer. Once we fully understand the mechanism of the binding between HLA-G and its receptors, effective inhibitors interfering with this interaction can be developed.

### 4.3. Functions of HLA-G

Studying all the functional aspects of HLA-G is essential in predicting how HLA-G-based immunotherapy will affect tumor growth. Functional assays have demonstrated that both soluble and membrane-bound HLA-G isoforms were able to inhibit multiple immune cell types [18]. For instance, NK cells showed reduced cytotoxicity upon binding HLA-G via the ILT2 or ILT4 receptors [73,74]. Furthermore, maturation and differentiation of DCs expressing ILT4 are inhibited by HLA-G [75]. Binding of HLA-G to ILT4 expressed by DCs led to a decreased antigen presentation, thereby disturbing communication between DCs and other immune cells. In addition, HLA-G was shown to induce apoptosis in CD8+ T cells and reduces proliferation of CD4+ T cells and B cells [12,76,77,78]. Thus, HLA-G can interfere in many different immunological processes of both the innate and adaptive immune system leading to a reduced immune response.

While immune cells are inhibited when HLA-G binds to its receptors, tumor cells might profit from the expression of both HLA-G and its receptors. Studies in patients with non-small lung carcinoma (NSCLC), gastric cancer, and CRC reported co-expression of HLA-G and its receptors ILT2 or ILT4 on tumor cells, and showed a correlation between co-expression and poor clinical outcome [79,80,81]. In addition, the studies in CRC and NSCLC patients analyzed the downstream pathways in cancer cells upon binding of HLA-G with the ILT4 receptor and showed increased phosphorylation of the ERK and AKT signaling pathways [79,81]. Furthermore, interaction between HLA-G and the ILT4 receptor on CRC and NSCLC cells resulted in the proliferation, migration and invasion of these cells, thereby promoting tumor progression [79,81]. The interaction between HLA-G and ILT4 resulted in upregulation of vascular endothelial growth factor-C (VEGF-C) and B7-H3 expression in NSCLC patients via the ERK and PI3K/AKT/mTOR signaling pathways [82,83]. B7-H3 is a member of the B7 family which is implicated in tumor progression by inhibiting T cell function, and by promoting the transformation of monocytes into tumor-associated macrophages [82]. VEGF-C belongs to a family that promotes tumor progression via enhancement of cell proliferation, invasion and metastasis [83]. These observations imply that tumor cells can express both HLA-G and its receptors, thereby initiating tumor-promoting processes in neighboring tumor cells that are not immune-related. It is remarkable that the interaction between HLA-G and ILT2 or ILT4 in tumor cells results in tumor progression, while binding of HLA-G to ILT2 or ILT4 expressed by immune cells results in suppression of these immune cells. To inhibit tumor growth with immunotherapy targeting HLA-G, both HLA-G and its receptors need to be present on the cell surface of tumor cells. As mentioned previously, co-expression of HLA-G and ILT2 or ILT4 has been observed in CRC, NSCLC and gastric cancer [79,80,81]. Therefore, immunotherapy targeting HLA-G can have two potential effects as it can restore both anti-tumor functions of immune cells and inhibit tumor growth.

## 5. HLA-G as Target for Immune Checkpoint Inhibition in Cancer

As discussed in this review, HLA-G may be an important target for immune checkpoint inhibition in cancer. Unfortunately, existing monoclonal antibodies directed against HLA-G are not sufficient and can, therefore, not be used for therapeutic purposes. Firstly, the majority of the available antibodies only recognize one or two isoforms of HLA-G [12,13,14,15,16]. Table 2 summarizes all widely used HLA-G-recognizing antibodies with their specificity. Although HLA-G1 and HLA-G5 are the most studied isoforms, all HLA-G isoforms are capable of modulating the immune system [84]. Therefore, inhibition of all HLA-G isoforms expressed by tumor cells is crucial to abrogate the full function of HLA-G in cancer. This includes both inhibitory effects on immune cells and stimulatory effects on tumor cells. The antibodies 4H84 and MEM-G/1 do recognize all HLA-G isoforms, but are known to cross-react with HLA class I molecules [17,18]. Secondly, it is unsure whether these antibodies can block the binding of HLA-G with ILT2, ILT4 and KIR2DL4. Antibodies can directly block the interaction between HLA-G and its receptors by binding to the receptor binding site or indirectly through steric hindrance. To directly block interaction between HLA-G and its receptors, antibodies should be targeted at the α3 domain and β2M of HLA-G for ILT2 and ILT4, and at the α2 domain for KIR2DL4. Hindering the interaction between HLA-G and its three receptors with a single antibody is challenging. Therefore, a cocktail of different antibodies that individually target each receptor may be necessary in order to block the interaction of HLA-G with all its receptors. Direct blockade of HLA-G2 homodimers has been demonstrated for MEM-G/1, which blocks the binding site of HLA-G2 for ILT4, thereby preventing binding [19]. In contrast, MEM-G/9 and G233 bind to HLA-G1, but do not share the same binding site of ILT2 [19]. Whether these antibodies can prevent the binding, either directly or indirectly, needs to be explored further. Only antibody 87G is often used in in vitro and in vivo experiments to block HLA-G1. These experiments showed that cell lysis by NK cells was restored, implying that 87G can interfere in the interaction between HLA-G1 and its receptors [85,86]. For future research, it is essential to develop antibodies that recognize all HLA-G isoforms, and to reduce the cross-reactivity of HLA-G-recognizing antibodies with other proteins. Moreover, it should be tested if the antibodies are able to hinder the binding of HLA-G to ILT2, ILT4 and KIR2DL4.

Since this approach is ambitious, it is also essential that alternative approaches are explored to block the interaction between HLA-G and its receptors. It is important to realize that antibody-based immune checkpoint inhibitors have disadvantages, including high costs, instability, immunogenicity and poor tumor penetration [99,100,101]. Due to these disadvantages, development of immune checkpoint inhibitors has been shifted to low-molecular-weight molecules, such as small molecules and peptides. These molecules have improved permeability, stability and tissue penetration [100,101,102]. Moreover, they can be orally administrated and are less immunogenic [100,102]. Currently, development of small-molecule inhibitors is mainly focused on targeting PD-1 and its ligand PD-L1, where multiple compounds are in development and one small-molecule is already licensed [101]. However, designing small-molecule drugs is challenging due to the large surface area where the binding between the receptor and ligand takes place, the need for well-defined binding pockets, and the highly hydrophobic interaction between many receptors and their ligands (including between PD-1/PD-L1, and HLA-G/ILT2/4) [99,100,102]. Another approach is to target the mRNA of the *HLA-G* receptor with RNA interference (RNAi). This post-transcriptional regulation process is based on small regulatory RNAs that are homologous to the target mRNA and causes degradation of the mRNA leading to gene silencing [103]. Multiple studies have shown that transfection of HLA-G positive cell lines with either microRNA (miRNA), short hairpin RNA (shRNA) or small interference RNA (siRNA) led to reduced mRNA and protein expression of HLA-G [104,105,106,107]. The viability, migration and invasion of oral squamous cell carcinoma (OSCC) cells were reduced as a result of decreased expression of HLA-G after transfection with a miRNA mimic [108]. This suggests that RNAi can be used to reduce HLA-G expression on tumor cells and, subsequently, inhibit its tumor-promoting effects. A challenge with this method is the delivery of the small regulatory RNAs to achieve the optimal dosage for gene silencing [103]. Small-molecule drugs and RNAi are promising alternatives for monoclonal antibodies, but both methods are still in development and more research is required before they can be implemented in the clinic.

Although receptor binding sites of HLA-G are the most obvious to target, other aspects of HLA-G can be targeted as well to diminish the function of HLA-G. We propose three alternative strategies that can be explored to interfere in the interaction between HLA-G and its receptors. First of all, dimerization of HLA-G can be prevented by blocking the cysteine residue at position 42 in the α1 domain. This can be achieved by targeting the α1 domain with antibodies that either directly block the cysteine residue, or sterically hinder dimerization. Almost all HLA-G isoforms are capable of forming dimers, thereby enhancing its inhibitory capacity. Various studies have shown that preventing dimerization can inhibit the function of proteins [109,110,111]. For example, Qi et al. demonstrated that inhibiting dimerization of the oncoprotein survivin leads to degradation of the protein and apoptosis in cancer cells [111]. Although the function of HLA-G will not be fully inhibited when dimerization is blocked, it may decrease the inhibitory capacity of HLA-G. This approach is only effective to decrease the interaction between HLA-G dimers and the ILT2 and ILT4 receptors since KIR2DL4 cannot bind HLA-G dimers.

Second, peptide presentation by HLA-G could be a potential target. Since the peptide presented by HLA-G does not contribute to the binding of HLA-G to its receptors, targeting the peptide binding groove for immune checkpoint blockade is challenging. However, the peptide binding groove can be used as target for immune checkpoint inhibition when a peptide could be developed with side chains large enough to cover the binding sites of HLA-G, thereby preventing receptor binding. Furthermore, due to the limited peptide repertoire and strong preference for particular peptide sequences, synthetic peptides specific for HLA-G may be produced conjugated to cytotoxic vehicles. Similar to antibody-drug conjugates (ADC), the cytotoxic vehicle is delivered at the cell surface via the synthetic peptide. The cytotoxic vehicle must be internalized to chemically kill the target cell. However, internalization of HLA-G is reduced when a high affinity peptide is loaded onto HLA-G, which is the case with synthetic peptides [112]. Alternatively, fluorochromes can be conjugated to the synthetic peptide to detect HLA-G in cells and tissues, thereby circumventing the use of specific HLA-G-recognizing antibodies. Though, cross reactivity with HLA-A2 can occur as the peptide profile of HLA-A2 and HLA-G1 overlap [38]. As mentioned above, the peptide repertoire of HLA-G seems to be dependent on the tissue it resides, which means that different synthetic peptides should be used for different tissues. Whether the peptide repertoire is similar between the different HLA-G isoforms is unknown since only peptides of HLA-G1 have been identified. This should be the focus of future studies.

Finally, another strategy is to target the HLA-G receptors with antibodies specifically binding to ILT2, ILT4 and/or KIR2DL4. This can effectively block the function of HLA-G as ILT2 and ILT4 bind with high affinity to HLA-G, and HLA-G appears to be the only ligand for KIR2DL4 [52,61]. The blockade can also restore the immunological function of immune cells as a recent study has shown that blocking ILT2 with an antibody resulted in increased tumoricidal activity of NK cells against various types of cancers [113]. However, ILT2 and ILT4 are known to bind to other classical and nonclassical HLA molecules as well, including HLA-A, -B, -C, -E and -F [114]. The inhibitory function of ILT2 and ILT4 is important for the maintenance of self-tolerance and is involved in neurological, developmental, and infectious responses [57]. Defective expression and function of ILT2 is associated with the autoimmune disease systemic lupus erythematosus [115]. Consequently, inhibiting ILT2 and ILT4 can lead to autoimmunity. The interaction between HLA-G and ILT2, ILT4 and KIR2DL4 is especially crucial in pregnant women, where it suppresses the maternal immune response at the fetal-maternal interface [116]. Considering the broad and location-specific functions of HLA-G receptors, it is desired to only inhibit these receptors in the tumor microenvironment (TME), where the receptors mediate immune evasion. This can be achieved by novel strategies that are developed to improve efficacy and safety of immune checkpoint inhibitors in cancer. For instance, an emerging strategy is the use of prodrug-formulated antibodies. These antibodies have a masking peptide that binds to a peptide binding site of the target receptor [117]. The masking peptide is cleaved by tumor-associated proteases in the TME and, as a result, the antibody is released to bind the target antigen [117,118]. HLA-G tumor expression could function as targeting molecule for such antibodies. Through this mechanism, the drug remains inactive when it binds to HLA-G in healthy tissue and is only activated when it binds to HLA-G in the TME [119]. This approach is dependent on the presence of tumor-associated proteases. It has been observed that HLA-G expression in ovarian cell line upregulates matrix metalloproteinase 15 (MMP-15) expression in these cells and a correlation between HLA-G and MMP-15 expression is also seen in ovarian cancer patients [120]. MMPs are suitable proteases to cleave prodrugs, so HLA-G-expressing tumors may be appropriate targets for prodrug-formulated antibodies [119]. A prodrug against HLA-G and its receptors can be developed by designing a masking peptide that binds in the peptide binding groove of HLA-G1 and linking this masking peptide to antibodies targeting ILT2, ILT4 or KIR2DL4, or HLA-G itself. As a result, the receptors will only be blocked when HLA-G is expressed by the tumor. However, the disadvantages and challenges of targeting the peptide binding groove of HLA-G that were mentioned previously should be taken into account with this approach as well.

In summary, targeting HLA-G with monoclonal antibodies or synthetic peptides to block the interaction with its receptors remains a challenge. Using the peptide binding groove as target for the binding of immune checkpoint inhibitors is promising, but more research has to be performed to further elucidate the peptide preferences of HLA-G to prevent cross reactivity and to cover all the isoforms of HLA-G.

## 6. Conclusions

HLA-G is considered an important immune checkpoint in cancer due to its strong immune-inhibiting functions, and thereby, it is able to facilitate immune escape and tumor growth. In addition, co-expression of HLA-G and its receptors has been observed in different cancer types, leading to proliferation, migration and invasion of tumor cells upon interaction. Hence, HLA-G can also initiate not immune-related tumor-promoting processes in neighboring tumor cells. Therefore, HLA-G is a potential target for immunotherapy to treat cancer. In this review, we discussed the structure of HLA-G and its receptors ILT2, ILT4, and KIR2DL4, and their interaction. In addition, we discussed the role of peptides presented by HLA-G in the binding of HLA-G to its receptors. Based on our findings, we propose three alternative strategies for HLA-G-based immunotherapy: (1) prevention of HLA-G dimerization, (2) targeting the peptide-binding groove of HLA-G, and (3) targeting the HLA-G receptors. Future research should focus on exploring these alternative strategies. Importantly, the function of HLA-G depends on the tissue where it is expressed and the type of cell that expresses its receptors. The contribution of HLA-G in the pathogenesis of different cancers should therefore be taken into account in future studies. This will aid in the selection of patients who will receive and benefit the most from HLA-G-based immunotherapy.

## Figures and Tables

**Table 1:** Primary structure and characteristics of peptides presented by HLA-G1. Each peptide position has a preference for certain amino acids and is either located in a binding pocket (A-F, further explained in Figure 2) in the peptide binding groove of HLA-G1, or is solvent exposed.

Peptide Position	Preferred Residue	Type of Amino Acid	HLA-G Binding Pocket	Characteristics
P1	Lysine (K)	Positively charged; aliphatic, hydrophilic	A pocket (conserved); solvent exposed	Auxiliary and tissue-specific anchor point; stabilizes binding
	Arginine (R)			
P2	Glycine (G)	Hydrophobic; aliphatic; amide group	B pocket	Debatable anchor point
	Isoleucine (I)			
	Leucine (L)			
P3	Proline (P)	Small; hydrophobic	D pocket	Primary anchor point
P4	Alanine (A)	Variable	Solvent exposed	Stabilizes binding
	Proline (P)			
P5	Alanine (A)	Variable	Highly solvent exposed	
	Arginine (R)			
	Glutamine (Q)			
P6	Alanine (A)	Hydrophobic; aromatic	C pocket	
	Phenylalanine (F)			
	Tyrosine (Y)			
P7	Isoleucine (I)	Small; hydrophobic	E pocket	
	Leucine (L)			
	Valine (V)			
	Tyrosine (Y)			
P8	Isoleucine (I)	Variable	Highly solvent exposed	
	Methionine (M)			
	Threonine (T)			
	Glutamine (Q)			
P9	Leucine (L)	Hydrophobic; aliphatic	F pocket (conserved)	Primary anchor point

**Table 2:** Overview of HLA-G-recognizing antibodies and their specificity.

HLA-G mAbs	Specificity	Reference
4H84	An α1 epitope in HLA-G	McMaster et al., 1998 [87]
MEM-G/1	Denatured free heavy chains of all HLA-G isoforms	Hurks et al., 2001 [88]
MEM-G/2	Free heavy chains of all HLA-G isoforms	Polakova et al., 2003 [89]
MEM-G/4	Free heavy chains of HLA-G1, -G2 and -G5 isoforms	Menier et al., 2003 [16]
MEM-G/9	HLA-G1 and -G5 associated with β2M	Fournel et al., 2000 [90]
MEM-G/11	HLA-G1	Boyson et al., 2002 [91]
MEM-G/13	HLA-G1 and -G5	Menier et al., 2003 [16]
G233	HLA-G1 and -G5	Loke et al., 1997 [92]
87G	HLA-G1 and -G5	Ødum et al., 1991 [93]
01G	HLA-G1	Real et al., 1999 [94]
BFL.1	HLA-G1	Bensussan et al., 1995 [95]
2A12	HLA-G5 and -G6	White et al., 2010 [96]
5A6G7	HLA-G5 and -G6	Le Rond et al., 2004 [97]
16G1	HLA-G5 and -G6	Blaschitz et al., 2000 [98]

Abbreviations: β2-microglobulin (β2M), human leukocyte antigen G (HLA-G), monoclonal antibodies (mAbs).
